# Assessment of delayed tuberculosis diagnosis preceding diagnostic confirmation among tuberculosis patients attending Isiolo County level four hospital, Kenya

**DOI:** 10.11604/pamj.2021.38.51.21508

**Published:** 2021-01-18

**Authors:** David Majuch Kunjok, John Gachohi Mwangi, Susan Mambo, Salome Wanyoike

**Affiliations:** 1 Jomo Kenyatta University of Agriculture and Technology (JKUAT), Nairobi, Kenya,; 2 Jomo Kenyatta University of Agriculture and Technology (JKUAT), College of Health Sciences (COHES), School of Public Health (SoPH), Nairobi, Kenya,; 3 Washington State University, Global Health, Nairobi, Kenya,

**Keywords:** Kenya, Isiolo, tuberculosis, delayed diagnosis, level four hospital, diagnostic confirmation

## Abstract

**Introduction:**

delayed diagnosis of Mycobacterium tuberculosis infection leads to accelerated individual to individual transmission. This study evaluated this aspect of delayed diagnosis among patients visiting Isiolo level four hospital in northern Kenya.

**Methods:**

this was a cross-sectional cohort study conducted during January, 2018-January, 2019 with systematically sampled 172 tuberculosis (TB) patients. Epidemiological and clinical characteristics were abstracted from records to serve as independent variables. Outcome variable was delayed diagnosis dichotomised into < 21 or > 21 days and treated as a binary outcome. Pre-tested interviewer-administered questionnaires, focused group discussions, and key informant interview guides were used to collect relevant information.

**Results:**

most (n=89, 57.8%) of the TB diagnosis fell in the category of > 21 day delay. Overall, among all patients, delay in days constituted a median of 27.6, a mean of 37.3 ± 57 days (range 0-414 days). Factors associated with delayed diagnosis (happening > 21 days) included (i) use of dispensary and private health facilities, (OR=4.3, 95% CI: 1.44,13.14; P=0.009) and (OR= 4.9, 95% CI: 1.64, 14.73; P=0.004), respectively (ii) Self-employed individuals (OR=21.7, 95% CI: 2.47,190.93; P=0.006) and employed individuals (OR=9.9, 95% CI: 1.14, 85.80; P=0.038) (iii) secondary-level education (OR=0.03, 95% CI: 0.01,0.21; P=0.000) and tertiary education (OR=0.033, 95% CI: 0.01, 0.23; P=0.001).

**Conclusion:**

delayed diagnosis of TB was found to be associated with health-seeking behaviour of TB patients, proxied by diagnosis facility, occupation, and education levels in our study area. Curtailment of local transmission of M. tuberculosis needs intensified health promotion and education in affected communities complemented with active case findings.

## Introduction

Tuberculosis is a respiratory infection caused by *Mycobacterium tuberculosis* bacteria and has been ranked seventh among other most important causes of death worldwide [1]. The pathogen infects the lungs (pulmonary TB) in addition to other structural parts of the human anatomy (extrapulmonary TB). Tuberculosis infection spreads among population by inhalation of bacterial droplets produced during coughing and sneezing by infected individual [2]. Tuberculosis is infecting millions of people every year [3]. In 2017, TB caused death of approximately 1.3 million HIV negative people globally [1]. The worldwide estimate of people who developed TB in 2017, according to WHO was 10.0 million of these, 5.8 million were men, 3.2 million women, and 1.0 million children. Africa accounts for 2.5 million TB infected individuals which is a quarter of overall new infections due to TB and it had 417,000 TB related deaths [2]. Even though sub-Saharan Africa makes up only 12% of the world population, it contributes 29% of all the TB cases worldwide and 254,000 TB-related deaths [4]. There is a rising trend of TB occurrence from the pre-survey assessment of 233 per 100,000 people in 2016 as equated to 558 per 100,000 people in 2017 in Kenya [1].

Kenya still has the highest-burden index of TB. There were about 169,000 TB infected individuals in 2016, and only 46% (77,376) were diagnosed and underwent treatment [1]. Eighty percent of the individuals who visit healthcare providers are not diagnosed at the first visit, while 23% are undiagnosed because they are asymptomatic and lack cardinal TB signs including weight loss, fever, drenching night sweat, and cough of more than two weeks [5]. Early diagnosis of TB could lead to effective control of the disease and also prevent its transmission to healthy individuals [6]. Diagnostic delay in patients is owed to health-seeking behavior, incorrect diagnostic procedures suggested by health care providers, and inadequate diagnostic capabilities at health care facilities [7]. This delay in TB diagnosis accelerates the transmission of the disease in the community, wherein untreated smear-positive patient infects an average of 15 people annually along with a negative impact on treatment outcome [8].

The time duration between the onset of TB-associated symptoms to the confirmation of the disease may contribute to delays in quest of care from the health care provider, and this increases the TB-related morbidity and mortality rates [9]. Late presentation by TB patients to the health providers is a significant problem in the developing world in which less than half of the predictable sputum smear-positive pulmonary tuberculosis is obtained [10]. Identification of TB cases is mostly passive, when patients visit health care providers with the symptoms of TB in Kenya [11]. This leads to the delay in diagnosis of TB. This study assessed the delayed TB diagnosis prior to confirmation and the factors for delay by determining the proportion of presentation of clinical symtpoms described in the WHO TB standard case definitions.

For study implementation, the standard WHO case definition for TB was employed to record the presented clinical symptoms mentioned in the patient´s record at the first visit to the health care facility up to the date of disease confirmation. The clinical symptoms included: cough of any duration, night sweat, fever, weight loss, chest pain, fatigue, productive cough, and shortness of breath. Specifically, the outcome variable was the interval, in days, between the first visit of patient to the health care facility when suggestive clinical symptoms of TB were experienced, and the time when the confirmed diagnosis of TB infection was done. Based on the diagnosis by the healthcare provider, this outcome variable was dichotomised into Delayed (> 21 days) and Not delayed (< 21 days). Many factors are known to contribute to delayed diagnosis of TB worldwide [12]. In this study, we assessed socio-demographic, socio-economic and clinical factors including age, sex, marital status, religion, history of TB in the family, family size, comorbidity, distance to a healthcare facility, type of facility for diagnosis, income level, self-medication, occupation, and place of residence and education level. Efforts to reduce the delayed TB diagnosis are of paramount importance for lowering the disease burden [11,13]. Developing novel approaches and strategies along with effective implementation of the existing information and data to reduce the TB incidence should be a public health priority, particularly in high-TB burden, resource-constrained countries such as Kenya. We assessed 172 TB patients with confirmed infection by microscopy, culture, or Gen Xpert who attended Isiolo County Level 4 hospital in northern Kenya from January 2018 to January 2019.

## Methods

**Figure 1:**
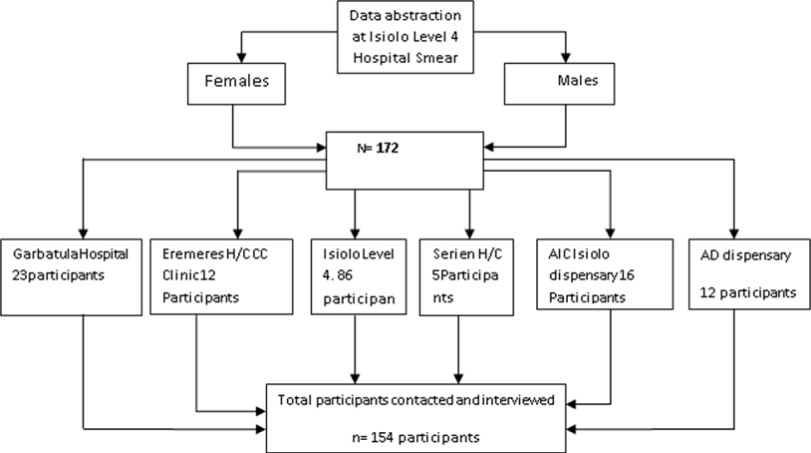
data collection protocol among study participants at Isiolo level 4 hospital, Isiolo County, Kenya 2019

The study was conducted at Isiolo County level four hospital in northern Kenya. The country has an estimated population of 143,294 people, according to the 2009 census. It borders Marsabit to the North, Samburu to the west, Mandera, Wajir, and Garissa to East, Tana River, Meru, and Laikipia to South and is comprised of 10 wards. The country was chosen for the study due to heavy burden of TB infection which is nearly 51% for every 10,000 people [14]. This was a cross-sectional cohort study and systematically sampled 172 TB patients were included using Yamane [15] formula and their epidemiological and clinical data was collected from January 2018 to January 2019. The TB patients referred from Isiolo level 4 hospital to their nearest health facility for treatment were contacted for an interview (Figure 1).

An abstraction form was used for collection of epidemiological and clinical data from the records. A pre-tested interviewer-administered questionnaire, focused group discussions, and key informants guides were used to collect the data and information regarding the factors associated with delayed TB diagnosis from the tuberculosis patients above five years of age who gave the consent for the same. Data was analysed using the SPSS version 20. The bivariate analysis was performed to obtain the descriptive statistics for socio-demographic, socio-economic, and clinical factors (signs and symptoms) at a level of significance of P-value of 0.2. We used a higher p-value not to leave out variables that might turn significant after controlling for confounding. The outcome variable was the interval, in days, between the first health facility visit of the patient when suggestive clinical symptoms of TB were experienced, and the time when a confirmed diagnosis of TB was made. To ensure comparability with other studies, we dichotomised our findings into delay (>21) days and no delay (≤ 21 days) according to WHO minimum cut-off days of the diagnosis. The variables included were those showing association at the p-value of ≤ 0.2 in bivariate analysis. Finally, we considered the strength of association to be statistically significant in the multivariable analysis at the level of significance of P <0.05. We screened plausible interactions in blocks for statistical significance (P <0.05) with TB delayed diagnosis using backward fitting logistic regression interaction. Multivariable regression analysis, which controlled for confounding, was used to determine the association between the screened independent variables at P-value of 0.2 and the dependent variable. The parsimonious model, which was run using the backward fitting logistic regression model, with a significant association, was selected.

## Results

**Proportion of clinical symptoms of TB among study participants:** most (n=148, 96.1%) of the study participants experienced a cough of diverse duration. Other clinical signs and symptoms included fatigue (n=153, 99.4%), chest pain (n=132, 85.7%), and fever (n=117, 76.0%). Shortness of breath (n=143, 92.9%), night sweats (n=142, 92.2%), and unexplained weight loss (n=147, 95.5%) were also noteworthy complaints. Most (n=98, 63.6%) of the study participants had other non-specific symptoms. A large majority (n=111, 72 .1%) of the participants were diagnosed with TB during their 2nd visit (Table 1).

**Table 1:** distribution of TB clinical signs and symptoms among study participants, Isiolo level 4 hospital, Kenya 2019

Variable	Variable Code	Frequency	Proportion (%)	95% CI of proportion
		n=154		
**No. of symptoms**				
0-1	0	115	74.7	67.0 - 81.3
2 or more	1	39	25.3	18.7 - 33.0
**Productive cough**				
Yes	0	148	96.1	91.7 - 98.6
No	1	6	3.9	1.4- 8.3
**Chest pain**				
Yes	0	132	85.7	79.2 - 90.8
No	1	22	14.3	9.2 - 20.8
**Night sweats**				
Yes	0	142	92.2	86.8 - 95.9
No	1	12	7.8	4.1 - 13.2
**Shortness of breath**				
Yes	0	143	92.9	87.6 - 96.4
No	1	11	7.1	3.6 - 12.4
**Unexpected weight loss**				
Yes	0	147	95.5	90.9 - 98.2
No	1	7	4.5	1.8 - 9.1
**Fever**				
Yes	0	117	76	68.4 - 82.5
No	1	37	24	17.5 - 31.6
**Fatigue**				
Yes	0	153	99.4	96.4 - 100.0
No	1	1	0.6	0.000 - 3.6
**Other symptoms**				
Yes	0	98	63.6	55.5 -71.2
No	1	56	36.4	28.8 - 44.5

**Socio-demographic and socio-economic characteristics of participants:** in this study, 172 respondents were included. Of these, 13 participants were relapse cases, follow up for one patient was lost, and 4 participants declined to give the consent. Overall,there were 154 participants, and the response rate was 89.5%. The median and mean age of the participants was 36 years (interquartile range 23-48) and 38 years, respectively. Most (n=84, 54.5%) of the respondents were male, (77, 50.0%) who belonged to the age group of 31 to 60 years. Over one-half of study participants (n=87, 56.5%) were Muslims, while the rest were Christians. The majority (n=97, 63%) of the study participants had primary school education, while 43 (28%) had secondary level of education. Over half of the study participants (n=88, 57.1%) were married. The distribution of occupation showed that less than half (n=52, 33.8%) of the participants were unemployed. Income status distribution had equal representation: (n=77, 50%) living on less than Ksh. 5700 p.m and the other half (n=77, 50%) on more than Ksh. 5700 p.m. Walking distance to the nearest healthcare facility was distributed with most of the participants (n= 99, 64.3%) having walking distance less than 5 km to reach the nearest healthcare facility. Less than half of the study participants (n=75, 48.7%) were from family sizes of between 3 and 5 individuals. Majority of (n=101, 65.6%) TB patients who participated in the study lived in the rural areas with few (n=53, 34.4%) participants from the urban areas (Table 2).

**Table 2:** distribution of socio-demographic, socio-economic and clinical characteristics among study participants, Isiolo level 4 hospital, Kenya 2019

Variable	Variable Code	Frequency	Proportion (%)	95% CI of proportion
		n=154		
**Age**				
Middle age (31-60yrs)	0	77	50	42.1 - 57.9
Young (7-30yrs)	1	56	36.4	28.8 - 44.5
Old (>60yrs)	2	21	13.6	8.6 - 20.1
**Sex**				
Male	0	84	54.5	46.3 - 62.6
Female	1	70	45.5	37.4 - 53.7
**Religion**				
Muslim	0	87	56.5	48.3 - 64.5
Christian	1	67	43.5	35.5 - 51.7
**Marital Status**				
Married	0	88	57.1	48.9 - 0.651
Single	1	44	28.6	21.6 -36.4
Widowed	2	13	8.4	4.6 - 14.0
Divorced	3	9	5.8	2.7 - 10.8
**Residence**				
Rural	0	101	65.6	57.5 - 73.0
Urban	1	53	34.4	27.0 - 42.5
**Education**				
Primary	0	97	63	54.8 - 60.0
Secondary	1	43	28	21.0 - 35.7
Tertiary	2	14	9	5.1 - 14.8
**Visit Diagnosis**				
2nd	0	111	72.1	64.3 -79.0
3rd	1	23	15	9.7 - 21.6
1st	2	15	9.7	5.6 - 15.6
4th	3	5	3.2	1.1 - 7.4
**Occupation**				
Unemployed	0	52	33.8	26.4 - 41.8
self-employed	1	48	31.2	24.0 - 39.1
Employed	2	42	27.3	20.4 - 35.0
Casual labourer	3	12	7.8	4.1 - 13.2
**Health Facility Of Dx**				
Public	0	56	36.4	28.8 - 44.5
Dispensary	1	54	35.1	27.6 - 43.2
Private	2	44	28.6	21.6 - 36.4
**Self-Medication**				
Yes	0	107	69.5	61.6 - 76.6
No	1	47	30.5	23.4 - 38.4
**Comorbidity**				
No	0	113	73.4	65.7 - 80.2
Yes	1	41	26.6	19.8 - 34.3
**Distance from HF**				
≤5Km	0	99	64.3	56.2 - 71.8
>5Km	1	55	35.7	28.2 - 43.8
Income				
≤KSh.5700	0	77	50	41.8 - 58.2
>KSh.5700	1	77	50	41.8 - 58.2
**History of TB in Family**				
No	0	105	68.2	60.2 - 75.4
Yes	1	49	31.8	24.6 - 39.8
**Family size**				
3 to 5	0	75	48.7	40.6 - 56.9
6 to 8	1	51	33.1	25.8 - 41.1
>8	2	15	9.7	5.6 - 15.6
0-2	3	13	8.4	4.6 - 14.0

**Heath seeking characteristics:** among the recruited participants, 56 were diagnosed for TB infection (36.4%) at the public healthcare facilities, 54 (35.1%) participants were diagnosed in the dispensary and diagnosis of 44 participants (28.6%) for TB was done at private healthcare facilities. Regarding participants´ self-medication, 107 participants (69.5%) attempted self-medication prior to the TB confirmation. The majority (n=113, 73.4%) of the participants had no comorbidities such as HIV, diabetes mellitus, hypertension, chronic obstructive pulmonary disease, asthma, and lung cancers. However, 41 participants (26.6%) reported having comorbidities. Most (n=105, 68.2%) of the study participants reported no family history of TB and 49 participants (31.8%) were with a positive family history of TB (Table 2).

**Figure 2:**
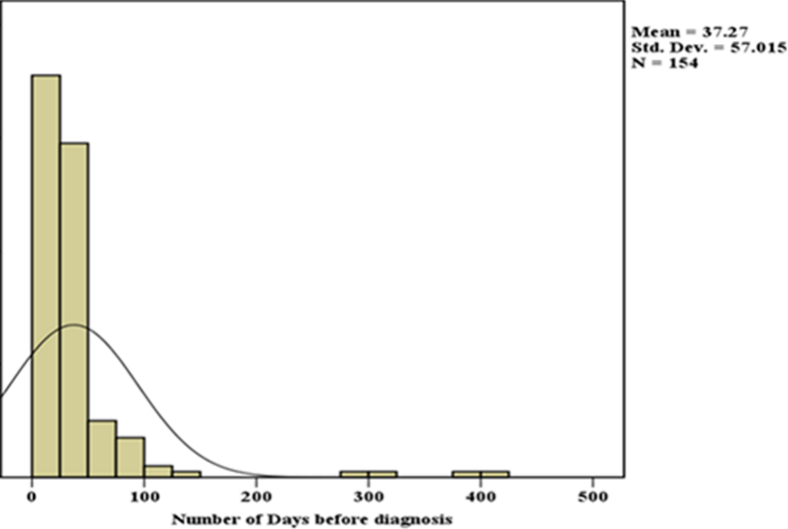
time interval to TB diagnosis among study participants, Isiolo County level 4 hospital Kenya 2019

**Time interval for TB diagnosis:** presentation of the clinical symptoms of TB resulted in the generation of trends on the time interval for TB diagnosis. Majority of the participants, i.e. 89 (57.8%) experienced diagnosis delays (>21 days), with a mean of 37.3 days (Table 3, Table 4). Only among 42.2% of the participants, diagnosis was not delayed, with the diagnosis being concluded in less than 21 days. The median number of days to TB diagnosis was 27.6 days, with the patients presenting for diagnosis at 0 days and the maximum days being 414 days (Figure 2).

**Table 3:** distribution of patients' signs and symptoms by the time interval to the diagnosis of TB in Isiolo level 4 hospital, Isiolo County, Kenya 2019

Variable	n=154(%)	Delayed Diagnosis of TB		Visit of Diagnosis	
		<21days	>21 days	<2nd visits	>2nd visits
**Productive cough**					
Yes	148(96.1)	62(41.9)	86(58.1)	129(87.2)	19(12.8)
No	6(3.9)	3(50.0)	3(50.0)	5(83.3)	1(16.7)
**Chest pain**					
Yes	132(85.7)	55(41.7)	77(58.3)	114(86.4)	18(13.6)
No	22(14.3)	10(45.5)	12(54.5)	20(90.9)	2(9.1)
**Night sweats**					
Yes	142(92.2)	57(40.1)	85(59.9)	123(86.6)	19(13.4)
No	12(7.8)	8(66.7)	4(33.3)	11(91.7)	1(8.3)
**Shortness of breath**					
Yes	143(92.9)	63(44.1)	80(55.9)	124(86.7)	19(13.3)
No	11(7.1)	2(18.2)	9(81.8)	10(90.9)	1(9.1)
**Unexpected weight loss**					
Yes	147(95.5)	60(40.8)	87(59.2)	127(86.4)	20(13.6)
No	7(4.5)	5(71.4)	2(28.6)	7(100)	0(0)
Fever					
Yes	116(75.3)	50(43.1)	66(56.9)	101(87.1)	15(12.9)
No	38(24.7)	15(39.5)	23(60.5)	33(86.8)	5(13.2)
**Other symptoms**					
Yes	98(63.6)	46(46.9)	52(53.1)	83(84.7)	15(15.3)
No	56(36.4)	19(33.9)	37(66.1)	51(91.1)	5(8.9)

**Table 4:** distribution of patients' socio-demographic, socioeconomic and clinical characteristics by the time interval to the diagnosis of TB among study participants, Isiolo level 4 hospital, Kenya 2019

Variable	n=154(%)	Delayed Diagnosis of TB		Visit of Diagnosis	
		<21days	>21 days	<2nd visits	>2nd visits
Total	154(100)	65(42.2)	89(57.8)	134(87.0)	20(13.0)
**Age**					
Middle age (31-60yrs)	77(50)	33(42.8)	44(57.1)	68(88.3)	9(11.7)
Young (7-30yrs)	56(36.4)	25(44.6)	31(55.4)	47(83.9)	9(16.1)
Old (>60yrs)	21(13.6)	7(33.3)	14(66.7)	19(90.5)	2(9.5)
**Sex**					
Male	84(54.5)	34(40.5)	50(59.5)	72(85.7)	12(14.3)
Female	70(45.5)	31(44.2)	39(55.7)	62(88.6)	8(11.4)
**Religion**					
Muslim	87(56.5)	44(50.6)	43(49.4)	75(86.2)	12(13.8)
Christian	67(43.5)	21(31.3)	46(68.7)	59(88.0)	8(12.0)
**Marital status**					
Married	88(57.1)	34(38.6)	54(61.4)	77(87.5)	11(12.5)
Single	44(28.6)	21(47.7)	23(52.3)	38(86.4)	6(13.6)
Widowed	13(8.4)	6(46.2)	7(53.8)	12(92.3)	1(7.7)
Divorced	9(5.8)	4(44.4)	5(55.6)	7(77.8)	2(22.2)
**Residence**					
Rural	101(65.6)	39(38.6)	62(61.4)	87(86.1)	14(13.9)
Urban	53(34.1)	26(49.1)	27(50.9)	47(88.7)	6(11.3)
**Education**					
Primary	97(63.0)	38(39.1)	59(61.0)	82(84.5)	15(15.5)
Secondary	43(28.0)	16(37.2)	27(62.8)	39(90.7)	4(9.3)
Tertiary	14(9.1)	11(78.6)	3 (21.4)	13(92.8)	1(7.2)
**Occupation**					
Unemployed	52(33.8)	33(63.5)	19(36.5)	41(78.8)	11(21.2)
self-employed	48(31.2)	19(39.6)	29(60.4)	44(91.7)	4(8.3)
Employed	42(27.3)	10(23.8)	32(76.2)	38(90.5)	4(9.5)
Casual labourer	12(7.8)	3(25)	9(75)	11(91.6)	1(8.3)
**Health Facility Of Dx**					
Public	56(36.4)	27(48.2)	29(51.8)	46(82.1)	10(17.9)
Dispensary	54(35.1)	28(51.9)	26(48.1)	48(88.9)	6(11.1)
Private	44(28.6)	10(22.7)	34(77.3)	40(90.9)	4(9.1)
**Self-Medication**					
Yes	107(69.5)	49(45.8)	58(54.2)	93(86.9)	14(13.1)
No	47(30.5)	16(34.0)	31(66)	41(87.3)	6(12.8)
**Comorbidity**					
No	113(73.4)	44(38.9)	69(61.1)	95(84.1)	18(15.9)
Yes	41(26.6)	21(51.2)	20(48.8)	39(95.1)	2(4.9)
**Distance from HF**					
≤5Km	99(64.3)	43(43.4)	56(56.6)	88(88.9)	11(11.1)
>5Km	55(35.7)	22(40)	33(60)	46(83.6)	9(16.4)
**Income**					
≤KSh.5700	77(50)	40(51.9)	37(48.1)	62(80.5)	15(19.5)
>KSh.5700	77(50)	25(32.5)	52(67.5)	72(93.5)	5(6.5)
**History TB in Family**					
No	105(66.2)	45(42.9)	60(57.1)	95(90.5)	10(9.5)
Yes	49(31.8)	20(40.8)	29(59.1)	39(79.6)	10(20.4)
**Family size**					
3 to 5	75(48.7)	29(38.7)	46(61.3)	69(92)	6(8)
6 to 8	51(33.1)	22(43.1)	29(56.9)	43(84.3)	8(15.7)
>8	15(9.7)	7(46.7)	8(53.3)	12(80)	3(20)
0-2	13(8.4)	7(53.8)	6(46.2)	10(76.9)	3(23.1)

**Patient factors associated with the delay of diagnosis for TB (P-value 0.05):** compared to Muslims, Christians had 2.3 odds (95% CI: 0.95, 5.34; P = 0.066) of delayed diagnosis. Compared to the non-specific symptoms, the absence of non-specific symptoms was 2.2 times (95% CI: 0.92, 5.44; P=0.076), more likely to lead to delayed diagnosis of TB. Self-employed individuals were 21.7 times (95% CI: 2.47, 190.93; P= 0.006) more likely to have delayed diagnosis of TB compared to employed individuals (OR=9.995% CI: 1.14,85.80; P=0.038). A casual laborer was 2.2 times (95% CI: 0.24, 19.71; P=0.490), more likely to have delayed diagnosis of TB compared to unemployed individuals. Diagnosis at a dispensary and private healthcare facilities was 4.3 times (95% CI: 1.44, 13.14; P=0.009) and 4.9 times (95% CI: 1.64, 14.73; P=0.004), respectively, more likely to have delayed TB diagnosis compared to the diagnosis at the public hospital. Individuals with income of > Kshs. 5700 were 2.0 times (95% CI: 0.83, 4.92; P=0.119) more likely to have delayed diagnosis of TB compared to study participants with an income level of < Ksh. 5700 (Table 5, Table 6).

**Table 5:** distribution of socio-demographic, socioeconomic characteristics, and health-seeking behaviour among TB patients Isiolo County level 4 hospital Kenya, 2019 (bivariate table)

Variable			B	S.E.	Wald	df	Sig.	OR	95% CI for OR	
	Variable Code								Lower	Upper
**Age**										
Middle age (31-60yrs)	0		Ref.							
Young (7-30yrs)	1		0.41	0.52	0.62	1	0.43	1.5	0.54	4.13
Old (>60yrs)	2		0.48	0.54	0.79	1	0.37	1.61	0.57	4.61
**Sex**										
Male	0		Ref.							
Female	1		-0.16	0.33	0.23	1	0.63	0.86	0.45	1.626
**Religion**										
Muslim	0		Ref.							
Christian	1		0.81	0.34	5.65	1	0.017*	2.24	1.15	4.36
**Marital status**										
Married	0		Ref.							
Single	1		-0.24	0.71	0.12	1	0.73	0.79	0.19	3.14
Widowed	2		0.13	0.74	0.03	1	0.86	1.14	0.27	4.83
Divorced	3		0.07	0.87	0.01	1	0.94	1.07	0.19	5.91
**Residence**										
Rural	0		Ref.							
Urban	1		-0.43	0.34	1.55	1	0.21	0.65	0.33	1.28
**Education**										
Primary	0		Ref.							
Secondary	1		-1.74	0.68	6.47	1	0.011*	0.18	0.05	0.67
Tertiary	2		-1.82	0.72	6.34	1	0.012*	0.16	0.04	0.67
**Occupation**										
Unemployed	0		Ref.							
self-employed	1		1.65	0.73	5.17	1	.023*	5.21	1.26	21.63
Employed	2		0.68	0.73	0.86	1	0.35	1.97	0.47	8.20
Casual labourer	3		-0.07	0.76	0.01	1	0.93	0.94	0.21	4.15
**Health Facility Of Dx**										
Public	0		Ref.							
Dispensary	1		1.15	0.45	6.61	1	.010*	3.17	1.32	7.62
Private	2		1.29	0.45	8.27	1	.004*	3.66	1.51	8.87
**Self-Medication**										
Yes	0		Ref.							
No	1		0.49	0.36	1.83	1	.176*	1.64	0.80	3.34
**Comorbidity**										
No	0		Ref.							
Yes	1		-0.49	0.37	1.84	1	.174*	0.61	0.29	1.25
**Distance from HF**										
≤5Km	0		Ref.							
>5Km	1		0.14	0.34	0.17	1	0.68	1.15	0.59	2.25
**Income**										
≤KSh.5700	0		Ref.							
>KSh.5700	1		0.81	0.33	5.90	1	.015*	2.25	1.17	4.32
**History TB in Family**										
No	0		Ref.							
Yes	1		0.08	0.35	0.06	1	0.81	1.09	0.55	2.17
**Family size**										
3 to 5	0		Ref.							
6 to 8	1		-0.62	0.61	1.036	1	0.31	0.54	0.17	1.77
>8	2		-0.43	0.62	0.48	1	0.49	0.65	0.19	2.21
0-2	3		-0.29	0.76	0.14	1	0.71	0.75	0.17	3.33

**Table 6:** distribution of socio-demographic, socioeconomic, and clinical characteristics among study participants, Isiolo level 4 hospital, Kenya 2019 (multivariate table)

Variable	Variable Code	B	S.E.	Wald	P	OR	95% CI of OR	
							Lower	Upper
**Religion**								
Muslim	0	Ref						
Christian	1	0.81	0.44	3.39	0.066	2.25	0.95	5.34
**Education**								
Primary	0	Ref						
Secondary	1	-3.45	0.97	12.6	0.000	0.032	0.01	0.21
Tertiary	2	-3.4	0.99	11.8	0.001	0.033	0.01	0.23
**Night Sweats**								
Yes	0	Ref						
No	1	-1.44	0.82	3.09	0.079	0.238	0.05	1.18
**Other Symptoms**								
Yes	0	Ref						
No	1	0.80	0.45	3.14	0.076	2.236	0.92	5.44
**Occupation**								
Unemployed	0	Ref						
Self-employed	1	3.08	1.11	7.71	0.006	21.72	2.47	190.93
Employed	2	2.29	1.10	4.31	0.038	9.87	1.14	85.80
Casual worker	3	0.78	1.13	0.48	0.49	2.17	0.239	19.705
**Health Facility**								
Public	0	Ref						
Dispensary	1	1.47	0.57	6.77	0.009	4.34	1.44	13.14
Private	2	1.59	0.56	8.1	0.004	4.91	1.64	14.73
**Comorbidity**								
No	0	Ref						
Yes	1	-0.66	0.48	1.91	0.167	0.515	0.20	1.32
**Income**								
≤KSh.5700	0	Ref						
>5700	1	0.706	0.453	2.43	0.119	2.025	0.83	4.92

**Protective factors for delayed diagnosis of TB:** secondary level of education (OR= 0.032, 95% CI: 0.01, 0.21; P=0.011) and tertiary education (OR= 0.033, 95% CI: 0.01, 0.23; p=0.012), respectively, were protective towards delayed diagnosis of TB in that order. Absence of night sweats (OR=0.238; 95% CI: 0.48,1.18; P=0.079) was less likely to have delayed diagnosis of TB. Absence of comorbidity was protective (OR=0.515, 95% CI: 0.20, 1.32; P=0.167) towards having delayed TB diagnosis.

### Testing for plausible interactions

**Interactions positively associated with delayed TB diagnosis:** interactions between education and gender (primary education vs. male) were 16.2 times (95% CI: 1.10, 237.53; P=0.042), more likely to delay diagnosis of TB. Interactions between gender and history of TB in the family (males vs. TB in the family) were 16.6 times (95% CI: 1.18, 234.05; P=0.038) likely to delay TB diagnosis. Interactions between religion and residence (Muslims vs. rural residence) were 141.3 times (95% CI: 1.28, 15,646.31; P=0.039) to delay TB diagnosis. Interactions between education and residence (primary education vs. rural residence) were 119.44 times (95% CI: 2.44, 5836.13; P=0.016) and interactions between residence and health-seeking practice (rural residence vs. self-medication) were 16.3 times (95% CI: 1.82, 144.76; P=0.012) to delay diagnosis of TB. Interactions between comorbidities and employment(co-morbidity vs. unemployed were 225.3 times (95% CI: 7.15, 7106.99; P=0.002) to delay diagnosis of TB. Interactions between married and fever were 128.9 times more likely to delay diagnosis of TB (95% CI: 1.82, 9142.57; P=0.025) (Table 7).

**Table 7:** socio-demographic, socio-economic, and clinical characteristics associated with delayed TB diagnosis, Isiolo County level 4 hospital, Kenya 2019 (parsimonious interaction model table)

Interaction	B	S.E.	Wald	df	Sig.	OR	95%CI of OR	
							Lower	Upper
Age * Health-facility	-1.98	1.00	3.91	1	0.048	0.14	0.01	0.98
Education * Sex	2.78	1.37	4.12	1	0.042	16.17	1.10	237.52
Sex * Occupation	-3.15	1.31	5.78	1	0.016	0.04	0.00	0.56
Sex * TB-in-family	2.81	1.35	4.33	1	0.038	16.59	1.18	234.04
Religion * Residence	4.95	2.40	4.25	1	0.039	141.25	1.28	15646.31
Marital-status * Fever	4.86	2.17	4.10	1	0.025	128.99	1.82	9142.57
Health-facility * Marital-status	-0.94	0.43	4.73	1	0.03	0.39	0.17	0.91
Education * Residence	4.78	1.98	5.81	1	0.016	119.44	2.44	5836.13
Residence * Occupation	-4.38	1.67	6.85	1	0.009	0.01	0.00	0.33
Residence * Self-medication	8.25	2.88	8.18	1	0.004	3819.21	13.42	1087080.30
Fever * Income	-4.05	1.95	4.3	1	0.038	0.02	0.00	0.80
Other-symptoms * Self-medication	-6.49	2.38	7.43	1	0.006	0.00	0.00	0.16
No. symptoms * Other-symptoms	3.55	1.72	4.26	1	0.039	34.69	1.19	1007.31
Comorbidity * Occupation	5.41	1.76	9.47	1	0.002	225.35	7.15	7106.99
Comorbidity * Income	-8.79	3.12	7.93	1	0.005	0.00	0.00	0.07
Education	-3.07	1.56	3.87	1	0.049	0.05	0.00	0.99

**Interactions negatively associated with delayed TB diagnosis:** interactions between age and health facility of diagnosis (middle age; 31-60 years vs. public health facility) were 0.138 times less likely to delay diagnosis of TB (95% CI: 0.019, 0.98; P=0.048). Interactions between gender and employment (male vs. unemployed) were 0.043 times less likely to delay diagnosis of TB (95% CI: 0.01, 0.56; P=0.016). Interactions between healthcare facility of diagnosis and marriage (public facility vs. married) were 0.391 times less likely to delay TB diagnosis (95% CI: 0.17, 0.91; P=0.030). Interactions between residence and employment (rural residence vs. unemployed) were 0.013 times less likely to delay diagnosis of TB (95%CI: 0.00, 0.33; P=0.009). Interactions between TB symptoms and income level fever vs. lower-level income; ≤ KSh. 5700) were 0.017 times less likely to have delayed diagnosis of TB (95% CI: 0.00, 0.80; P=0.038). Interactions between non specific signs and symptoms of TB and health-seeking practice(fever vs. self-medication) were 0.002 times less likely (95% CI: 0.00, 0.16; P=0.006) to delay diagnosis of TB. Interactions between comorbidities and income level (Cormorbidity vs. lower income level; ≤ KSh. 5700) were 0.000 times less likely to delay diagnosis of TB (95% CI: 0.00, 0.07; P=0.005), (Table 7).

## Discussion

### Clinical symptoms listed in standard TB case definitions

The major clinical symptoms in this study included a cough lasting for more than three weeks, fatigue, chest pain, fever, shortness of breath, night sweats, and unexplained weight loss. Many of the study participants also had other non-specific symptoms.

The most shared clinical manifestation of TB included severe cough, fever, chest pains, fatigue, and weight loss [16]. Similarly, clinical signs such as persistent coughs lasting more than three weeks, chest pains, sweating at night, coughing blood or sputum, weight loss, lack of appetite, and fatigue have been reported [17]. Our findings contradicted other previous reports which revealed common symptoms related to the delay for TB diagnosis to be cough, weight loss, and loss of appetite [18]. A similar study conducted in Tanzania showed that TB diagnosis delay was more likely among patients who had no chest pain and who presented with hemoptysis [19]. Indeed, the participants indicated that these signs would disappear with time and, regrettably, they did not associate them with TB, perhaps escalating local transmission [20]. It is well established that patients with a productive cough lasting > 2 weeks are potential TB cases and should be subjected to microscopic examination or other investigations such as culture and Gene Xpert [21]. In our study settings, clinicians seemed to have low suspicion index of TB, perhaps compounded by inadequate laboratory diagnostic capacities and infrastructures, thus contributing to the delayed diagnosis of TB [22].

### Our outcome-the time interval between the first contact with the health care system and confirmed diagnoses

A thrust of this study was to assess the interval between the patient´s first contact with the health care system and confirmed diagnoses as a proxy of delay in diagnosis of TB, which we found to be highly heterogeneous. The median interval was 27.6 days, ranging between 0 and 414. Most of the diagnoses experienced delays of > 21 days, a period longer than the WHO recommendation [1]. These findings were comparable to the results of a study conducted in Dar es Salam, Tanzania, which reported a median delay of 3 weeks [19]. Another study in which a retrospective cohort approach was implemented evaluated the health-seeking behavior and extent of patient delays among TB patients and found a median patient delay of 20 days [23]. Our results were also comparable with the findings of the studies conducted in Brazil and Angola [12,24]. The median delay days reported in the Afar Region, Ethiopia, was 33.5 days, which was slightly different from our findings [25]. Noticeably, our study findings substantially differed from a study conducted in Huambo, Angola, which reported a median delay days as 64 [22]. Delay in diagnosis among patients in low and middle income countries varies from 4.9 days in Gambia to 162 days in Tanzania [26]. In the high income countries, this delay ranges from 7 days in Italy to 34.5 days in UK [27,28]. The average delay in low income and middle-income countries is found to be 28.4 days, while the high-income countries have reported delay in TB diagnosis of 21.5 days [29]. The reasons for this difference could be attributed to the better health services and inadequate health care services in high income and low or middle-income countries, respectively. Most of the patients were diagnosed with TB at a second visit to the health care provider. This finding is an indication that the diagnosis of TB was missed by the health care system in most of the patients at the initial visits [5]. This emphasises the need for evolving innovative ways focused on patients and the healthcare system to shorten these delays and, thereby, lessen burdens linked to the local transmission and adverse treatment outcomes.

A study carried on patients´ pathway [30], which showed that most of the TB patients initiated care at a low-level healthcare facility. These healthcare facilities did not have TB diagnostic capacity suggesting low suspicion of TB among communities alluded to the above. Private clinicians in rural areas which are hard-to-reach areas such as in our study run first line clinics broadly focusing on the provision of first aid services for simple ailments such as fever. Modalities should be designed targeting these clinics to encourage referrals of suspected cases showing TB flagship symptoms to government health centres [31].

### Factors associated with the time interval between the first visit to the health care system and confirmed diagnosis of TB

Our study showed that the level of education was associated with delayed diagnosis of tuberculosis. This finding was comparable to a study conducted in Bahir Dar City, Northwest Ethiopia [32], which indicated that uneducated patients were more likely to have delays in tuberculosis diagnosis. Moreover, delay in TB diagnosis was linked to several other factors which included socioeconomic status, patient´s awareness, and health system-related factors [33]. The study in Ethiopia also revealed different socioeconomic status and residence, religion, monthly income as independent factors for delay in TB diagnosis among patients [34]. Another study suggested that factors such as seeking treatment in low-level facilities are also associated with delays in tuberculosis diagnosis [35,36]. In this study, we evaluated the healthcare facility for diagnosis of TB and found its association with delayed diagnosis.

The primary education level was associated with longer diagnostics delays, and higher education level was considered an indicator of TB knowledge [37]. Our study found that being in the secondary and tertiary levels of education was protective for delayed diagnosis of TB. This study also revealed that those with primary education and residence in rural areas were prone to have delayed TB diagnosis. Besides, an interaction of primary education and male gender could lead to a higher chance of delayed TB diagnosis. The statistical association between education and delay in diagnosis needs to be explained because just having a certain level of education could not be a reason for one getting diagnosed within or beyond the recommended time. This implies that there might be an intervention of other factors between these two variables [37]. People with higher levels of education tend to have more knowledge and awareness about medical needs and also have access to better medical care than those with low education linked to the provision of higher-income skilled and low-income unskilled labour, respectively [37]. We theorised that health-seeking behaviour was an intervening variable in this statistical relationship between our outcome and education level.

Our study revealed that self-employed and employed individuals were associated with delayed diagnosis of TB. The likely reason is that self-employed and employed individuals could have a tendency for not wasting their precious income earning time for clinic visits [23,38,39]. Diagnosis of TB at a dispensary and private health facility was associated with delayed TB diagnosis in this study, and this is suggestive of a lack of adequate diagnostic capacity, whether in private or in public health facilities which contribute to the TB diagnosis delays [40]. Private healthcare facilities were associated with delayed diagnosis of TB when compared to the public health facilities. This could be due to the ease of access of public health-care providers for diagnostic procedures at the centers and readily available awareness [41]. Private health-care workers do not have robust links with the public healthcare system. Lack of training among health-care providers in the private sector further contributes to the delay in diagnosis, and it may perhaps be due to recurrent change of doctors [42].

The shorter delays of a confirmed diagnosis of tuberculosis were more seen among patients with chronic illnesses like HIV [43]. This study showed the absence of comorbidities and lower-income (= KSh. 5700 or US$57 per month) at the same time as protective factor against delayed TB diagnosis. The likely explanation for this is that individuals who sought care at the public health facilities were people of lower-income level because at these centers the health care services were either subsidised or free of charge. Higher-level public health facilities have an excellent capacity to diagnose TB in time. Medical care seems to intercede between the income and delayed diagnosis of TB. People with high incomes lean towards better medical care than those with low incomes. The level of income is an essential factor in early TB diagnosis [34]. Individuals with less income were expected to have delayed TB diagnosis and treatment than those earning more [44]. However, this study revealed that individuals with the income > Kshs. 5700 or US$ 57 per month were more likely to have delayed diagnosis. Furthermore, this is because individuals of higher economic levels were more inclined to seek health care from private health facilities which were found to be one of the factors for delayed TB diagnosis. The reason for this being lack of awareness and suspicion of tuberculosis by clinicians and doctors as reported by Montenegro ministry of health [45]. People with high incomes tend to have better medical care than those with low incomes. In our study, medical care is an intervening variable which mediates the relationship between income and diagnosis delay. Our study did not disentangle patient´s and health system factors for delayed diagnosis of TB. We relied on patients to determine delays, which could be either underestimated or overestimated, thus could not generalize the results.

## Conclusion

Current TB management strategies rely on passive case detection, but our findings suggest that they are inadequate to reduce onward community transmission and overall incidence. Both patients and health care workers do not prioritise the signs and symptoms due to various factors including health facility factors, health-seeking behavior of the patient, and education level leading to the delays in diagnosis. Addressing these factors could accelerate reduction in the tuberculosis incidences and lead to local control of the infection transmission. Therefore, there is a need for promotion of the health education in the community, deliberate strengthening of health care workers' suspicion index of TB diagnosis in high burden settings, and implementing dedicated TB-specific public-private health facility linkages. Prospective studies are needed to disentangle determinants and their interactions linked to the delay in seeking health care (patient delay) or delay in confirmed diagnosis of TB (health system delays) in high TB burden settings.

### What is known about this topic

- Factors associated with delayed diagnosis of TB;
- The median delay in developing countries around 28 days;
- Healthcare system delays exist.

### What this study adds

- This study has provided evidence-based data to local policymakers which is specific to the resource setting and geographic region;
- The study employed established and emerging risk factors, hypothesising their roles as potential confounders and mediators for consideration in future epidemiological studies;
- Generated data can be used to model onward community transmission of TB attributable to delays in diagnosis.
